# Cancer Induces Cardiomyocyte Remodeling and Hypoinnervation in the Left Ventricle of the Mouse Heart

**DOI:** 10.1371/journal.pone.0020424

**Published:** 2011-05-26

**Authors:** Christian Mühlfeld, Suman Kumar Das, Frank R. Heinzel, Albrecht Schmidt, Heiner Post, Silvia Schauer, Tamara Papadakis, Wolfgang Kummer, Gerald Hoefler

**Affiliations:** 1 Institute of Anatomy and Cell Biology, Justus-Liebig-University Giessen, Giessen, Germany; 2 Institute of Pathology, Medical University Graz, Graz, Austria; 3 Division of Cardiology, Medical University Graz, Graz, Austria; University of Padova, Italy

## Abstract

Cancer is often associated with cachexia, cardiovascular symptoms and autonomic dysregulation. We tested whether extracardiac cancer directly affects the innervation of left ventricular myocardium. Mice injected with Lewis lung carcinoma cells (tumor group, TG) or PBS (control group, CG) were analyzed after 21 days. Cardiac function (echocardiography), serum levels of TNF-α and Il-6 (ELISA), structural alterations of cardiomyocytes and their innervation (design-based stereology) and levels of innervation-related mRNA (quantitative RT-PCR) were analysed. The groups did not differ in various functional parameters. Serum levels of TNF-α and Il-6 were elevated in TG. The total length of axons in the left ventricle was reduced. The number of dense core vesicles per axon profile was reduced. Decreased myofibrillar volume, increased sarcoplasmic volume and increased volume of lipid droplets were indicative of metabolic alterations of TG cardiomyocytes. In the heart, the mRNA level of nerve growth factor was reduced whereas that of β1-adrenergic receptor was unchanged in TG. In the stellate ganglion of TG, mRNA levels of nerve growth factor and neuropeptide Y were decreased and that of tyrosine hydroxylase was increased. In summary, cancer induces a systemic pro-inflammatory state, a significant reduction in myocardial innervation and a catabolic phenotype of cardiomyocytes in the mouse. Reduced expression of nerve growth factor may account for the reduced myocardial innervation.

## Introduction

Cancer cachexia is a complex syndrome clinically manifesting as progressive loss of body weight with or without decreased food intake, and it is correlated with a poor prognosis [1]. The pathological involvement of the heart under these conditions was described as a new entity by Burch et al. [2] and termed the “cachectic heart”. Besides changes in the ECG and decreased heart size in chest x-rays, the cachectic heart is characterized by loss of epicardial fat, increase in lipofuscin granules and decrease in cardiomyocyte cross-sectional area despite generally normal cardiac function [3], [4]. Additionally, protein mass is decreased resulting from increased protein catabolism [5].

Interestingly, cancer is associated with functional alterations of the cardiovascular system, such as decreased heart rate variability in acute leukemia patients [6], increased resting heart rate, decreased resting blood pressure and increased postural fall in blood pressure in bronchial carcinoma patients [7], and increased incidence of cardiovascular autonomic insufficiency as assessed by a variety of electrocardiographic and clinical tests in breast cancer patients [8], [9]. Recently, a link has been hypothesized between cancer fatigue syndrome (a combination of dyspnea, exercise limitation and muscle weakness) and clinically non-overt heart failure, suggesting the fatigue symptoms to arise from autonomic dysfunction [10]. Although these studies clearly point to an involvement of the cardiac innervation in cancer cachexia, systematic studies on this topic are lacking so far.

The innervation of the ventricles predominantly consists of postganglionic sympathetic axons although, to a minor extent, also sensory and postganglionic parasympathetic axons are present [11], [12]. At the light microscopic level, immunohistochemistry is needed to visualize the unmyelinated cardiac nerve fibers. Each nerve fiber may consist of one or more axons, the number of which can only be determined by electron microscopy. Besides the classical neurotransmitter noradrenaline, sympathetic neurons also contain neuropeptides that are produced in the perikarya and stored in vesicular structures that are termed large dense core vesicles (LDCV). LDCV are anterogradely transported through the axon and are released upon burst or high frequency firing [13]. In the case of cardiac sympathetic axons, LDCV predominantly contain neuropeptide Y (NPY) [14].

Here, we hypothesized that cancer cachexia is associated with qualitative and/or quantitative structural alterations of the myocardial innervation. In order to test this hypothesis, we used a mouse model of tumor cachexia and examined its characteristics with respect to serum cytokine levels and cardiac function. In this model, we performed a detailed light and electron microscopic analysis of the left ventricle and employed design-based stereological methods to quantify various characteristics of cardiomyocytes and their innervation. In addition, the mRNA expression levels of various proteins related to cardiac innervation were quantified in the heart and the stellate ganglion, a major ganglion supplying sympathetic fibres to the heart.

## Results

### Animals

From the time point of tumor implantation until the end of the experiment after 21 days, the TG mice lost 2.32±0.82 g of lean body weight while the mice in CG gained 2.11±0.37 g of lean body weight (p<0.001) validating the mouse model as a model of tumor cachexia. The tumors themselves had a mean weight of 3.3±0.57 g. There were no significant differences in the weight of the left ventricle between the groups, however, the ratio between left ventricle and body weight was significantly higher in TG due to the decreased body weight (**Table 1**).

**Table 1:** Body and tumor weight.

	Control group	Tumor group
Body weight at day 0 [g]	20.1±0.8	20.2±0.6
Body weight (without tumor) at day 21 [g]	22.2±0.9	17.9±1.0**
Tumor weight [g]	0	3.3±0.6***
Left ventricular weight [mg]	70.5±2.2	68.0±1.8
Left ventricle-to-body weight ratio [mg/g]	3.18±0.10	3.81±0.23**

Legend. Data are expressed as mean ± standard deviation.

* indicates p<0.05,

**indicates p<0.01,

***indicates p<0.001.

### Echocardiography

Table 2 shows the data obtained by echocardiography in anesthetized, normothermic mice. None of the parameters differed significantly between TG and CG indicating a preserved cardiac function in the tumor group at resting conditions.

**Table 2:** Echocardiographic data.

	Control group	Tumor group
HR [min^−1^]	425±52	401±76
FS [%]	41±5.4	44±6.1
LvedD [mm]	3.75±0.1	3.50±0.18
LvesD [mm]	2.21±0.25	1.97±0.26
PW [mm]	0.64±0.03	0.66±0.07
SW [mm]	0.68±0.03	0.65±0.10

Legend. Data are expressed as mean ± standard deviation. There were no significant differences between CG and TG. HR = heart rate, FS = fractional shortening, LvedD = left ventricular end-diastolic diameter, LvesD = left ventricular end-systolic diameter, PW = posterior wall thickness, SW = septum thickness.

### TNF-α and Il-6 levels

The serum levels of TNF-α and Il-6 were significantly elevated in TG compared to CG (Table 3).

**Table 3:** Serum levels of TNF- α and Il-6.

	Control group	Tumor group
TNF-α [pg/ml]	13.02±4.12	28.11±1.92***
Il-6 [pg/ml]	10.58±2.77	18.41±3.93**

Legend. Data are expressed as mean ± standard deviation.

**indicates p<0.01;

***indicates p<0.001.

### Quantitative RT-PCR

In the heart, the expression of the β1-adrenergic receptor was unchanged in TG vs. CG. In addition, the expression of nerve growth factor (NGF) was decreased in TG compared with CG. Expression levels of both TNF receptors (TNF-R1, TNF-R2) were increased threefold or more, respectively, in TG vs. CG.

In the stellate ganglia of TG, the expression of NPY was significantly reduced whereas the expression of tyrosine hydroxylase, the rate-limiting enzyme for noradrenalin synthesis, was increased threefold. NGF was significantly downregulated in tumor-bearing mice but the changes were less extensive than the changes in transmitter expression. Both TNF receptors were significantly enhanced in TG vs. CG (Table 4).

**Table 4:** Relative expression of various innervation-related targets.

	Control group	Tumor group
Heart		
TNF-R1	1±0.09	3.56±0.19***
TNF-R2	1±0.09	3.05±0.25***
β1-R	1±0.15	1.13±0.27
NGF	1±0.19	0.43±0.43*
Stellate ganglion		
TNF-R1	1±0.03	1.69±0.08***
TNF-R2	1±0.08	1.93±0.19***
NGF	1±0.2	0.61±0.09*
NPY	1±0.24	0.38±0.11**
TH	1±0.19	3.24±0.10***

Legend. Data are expressed as mean ± standard deviation.

*indicates p<0.05,

**indicates p<0.01,

***indicates p<0.001.

TNF-R1 = tumor necrosis factor receptor 1, TNF-R2 = tumor necrosis factor receptor 2, β1-R = β1 adrenergic receptor, NGF = nerve growth factor, NPY = neuropeptide Y, TH = tyrosine hydroxylase.

### Cardiomyocyte morphology

**Figure 1:**
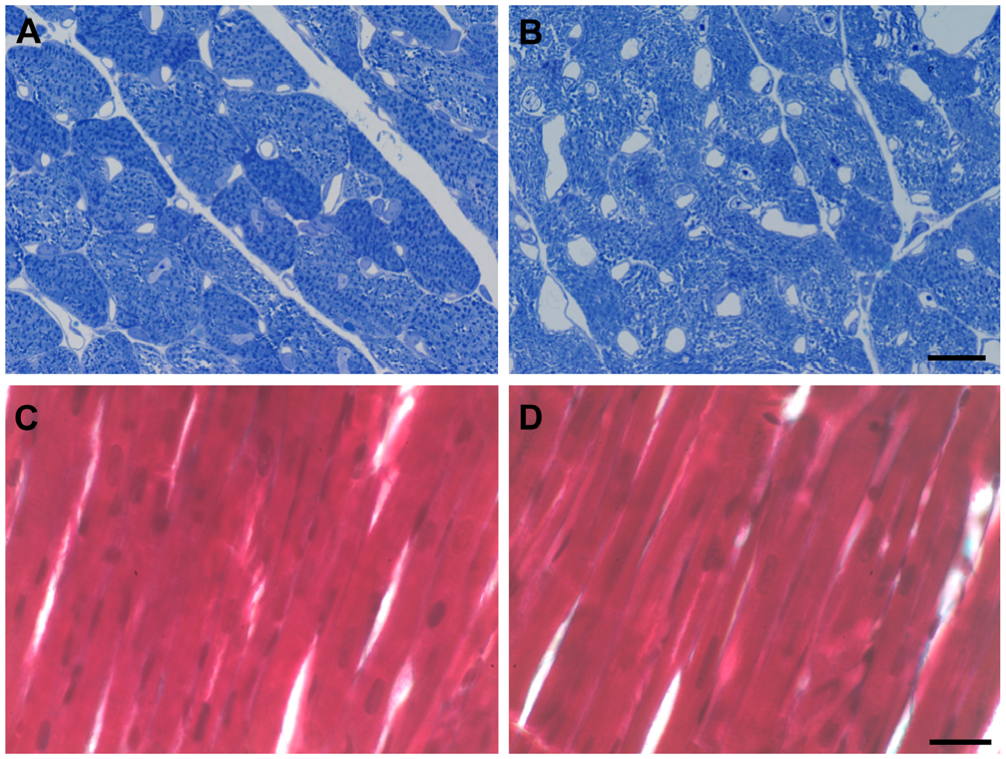
Morphology of myocardium. Semithin cross-sections (1 µm thickness) stained with toluidine blue did not show any differences in the dimensions of cardiomyocytes between control (A) and tumor-bearing mice (B). However, the subcellular organization of cardiomyocytes appeared less dense in the tumor group. Paraffin longitudinal sections (5–7 µm thickness) stained with Masson-Goldner's trichrome did not provide evidence for an enhanced collagen deposition in the tumor-bearing mice (D) as compared to control mice (C). Scale bar for A and B = 10 µm. Scale bar for C and D = 20 µm.

**Figure 2:**
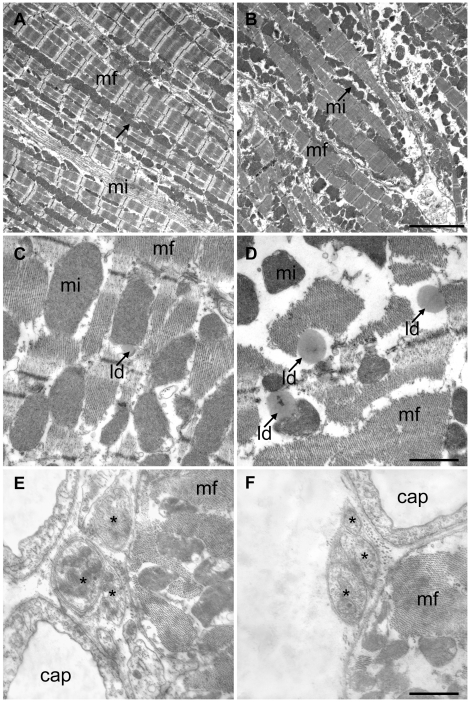
Ultrastructure of cardiomyocytes and nerve fibers. Electron micrographs of myocardium from control (A, C, E) and tumor-bearing mice (B, D, F). A and B demonstrate the the increased volume of sarcoplasm and the loss of myofibrils in more or less longitudinally sectioned myocytes. Scale bar = 5 µm. C and D clearly show the increase in lipid droplet (ld) size and number in the tumor group in diagonally sectioned myocytes. Scale bar = 1 µm. E and F show nerve fibres containing three axons (asterisk) each. There were no apparent differences in the morphology. The neighbouring myocytes are more or less transversely cut. Abbreviations: cap = capillary, mf myofibrils, mi = mitochondria.

At the light microscopic level, the myocardium appeared normal with well-preserved cardiomyocytes, widely opened capillaries due to perfusion fixation and smooth connective tissue in the interstitium. There were no signs of cardiac atrophy or interstitial fibrosis. This observation was supported by the total volume and number of cardiomyocytes which did not differ significantly between the groups (**Figure 1**). At the electron microscopic level, the two groups showed remarkable differences with respect to their myofibrils and their sarcoplasm. As depicted by the representative micrographs and the stereological data, the volume fraction of myofibrils was significantly reduced whereas the sarcoplasmic volume fraction was significantly enhanced in TG (**Figure 2**). The absolute volume of sarcoplasm was significantly enhanced whereas the decrease in the total myofibril volume failed to reach statistical significance (p = 0.06), probably because other factors like cardiomyocyte volume and left ventricular volume introduce a higher variability into the data. No differences were observed in the nuclear and mitochondrial volume density or volume (data not shown). Furthermore, in TG a higher number of larger sized lipid droplets in the sarcoplasm were noticed which is reflected by a higher total volume of lipid droplets in the left ventricle (Figure 2). This increase in lipid droplets, however, is far too low to explain the increase in total sarcoplasmic volume.

### Lipotoxicity

The biochemical analyses of the triglyceride content confirmed the increase in lipids in cardiomyocytes. Malondialdehyde and other thiobarbituric acid reactive substances are highly reactive compounds that are used as markers for oxidative stress. Using two tests for measuring these substances, we did not find a significant difference between CG and TG (**Table 5**). The end point of the toxic action of lipids in the heart is cardiomyocyte apoptosis. However, caspase activity did not differ between the groups (Table 5). Additionally, we did not find any morphological evidence for cardiomyocyte apoptosis by electron microscopy.

**Table 5:** Lipid content, lipid peroxidation and caspase 3/7 activity.

	Control group	Tumor group
Triglycerides per unit myocardium [µg/mg]	12.12±3.75	19.5±7.91*
MDA adduct per unit protein [pmol/mg]	3.25±0.47	3.77±0.63
TBARS per unit protein [µM/mg]	1.44±0.49	2.04±0.73
Caspase 3/7 activity per unit protein [LU/mg]	77.8±11.4	91.8±14.6

Legend. Data are expressed as mean ± standard deviation. MDA = malondialdehyde, TBARS = thiobarbituric acid reactive substances, LU = luminescence units.

* = p<0.05.

### Total length and morphology of myocardial axons

**Figure 3:**
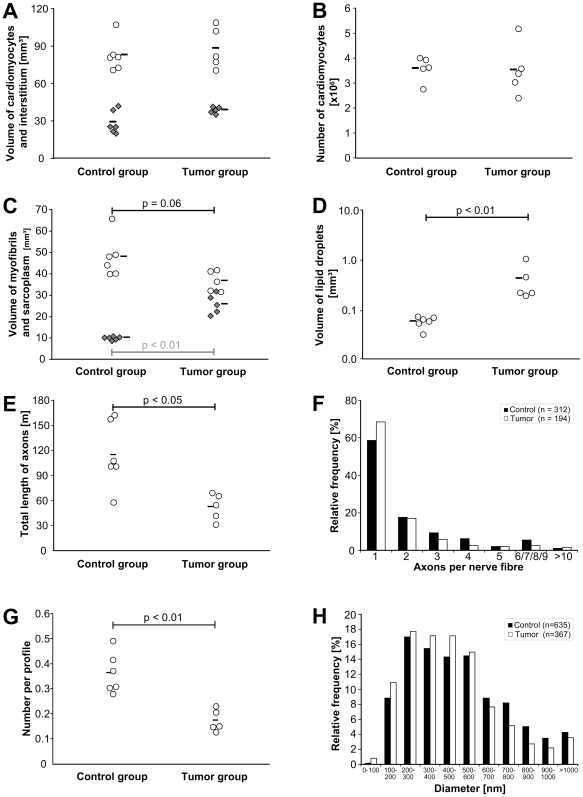
Stereological results. Neither the volume of cardiomyocytes (A, open circles) nor the volume of the interstitial space (A, filled squares) was different between the groups. In addition, the number of cardiomyocytes was similar among the groups (B). The volume of sarcoplasm (C, filled squares)was significantly increased in the tumor group while the volume of myofibrils (C, open circles) tended to be smaller. A greater volume of lipid droplets was found in the cardiomyocytes of tumor-bearing than of control mice (D). Note the logarithmic scale on the y-axis (D). The total length of axons was reduced to approximately one half in the tumor group (E) while the relative frequency of nerve fibers with a certain number of axons did not differ between the groups (F). The number of LDVC per axon profile was reduced in the tumor group (G) while the diameter of the axons was similar in both groups (H). Each circle or square represents the data for an individual animal. The horizontal bars demonstrate the group mean.

Figure 3 shows the morphology of the myocardial innervation and the stereological data of the axon characterization. The total length of axons ramifying between the cardiomyocytes was reduced to less than one half in TG when compared to CG. However, the relative frequency of nerve fibers with a particular number of axons did not vary between the groups. There were also no differences in the size of nerve fiber diameters although there was a tendency to a more frequent occurrence of axons with smaller diameters in the tumor mice. Interestingly, the number of neuropeptide storing LDCV per axon profile were reduced by about 50% in TG compared with CG.

## Discussion

The present study tested the hypothesis that chronic exposure to a large extracardiac tumor causes structural and functional alterations of the myocardial innervation in mice. The hypothesis was based on several reports on functional changes of the heart in patients suffering from a variety of forms of cancer [6]–[9] and the observation of degenerative structural lesions of cardiomyocytes in cancer cachexia [2]. In agreement with our hypothesis, we observed a pronounced reduction of the total length of axons innervating left ventricular cardiomyocytes and the number of neuropeptide storing organelles, LDCV, in these axons. Furthermore, we observed a decrease of myofibrillar volume but increased sarcoplasm and lipid droplet volume inside cardiomyocytes. The structural alterations were accompanied by reduced expression levels of NGF in heart and stellate ganglion, reduced ganglionic NPY expression but enhanced tyrosine hydroxylase expression.

The mouse model of Lewis lung carcinoma used in this study has been well-established for many years [15] and is known to cause a significant tumor cachexia [16]. In this study, the time between implantation of tumor cells and sacrifice of the animals was 21 days; this duration was sufficient to induce a tumor burden of 12–15% of total body weight and a cachectic condition simulating the clinical scenario of cancer cachexia. Despite the changes in body mass the hearts of TG mice had a similar wet mass as those of CG mice which contrasts with other studies using solid tumors [17]. These differences may be due to lower tumor burden, duration of the experiment, mouse strain or tumor cell type. As discussed below, the stereological results clearly indicate a catabolic phenotype of the cardiomyocytes although cardiac atrophy in the sense of reduced mass did not occur. The functional performance of the heart was assessed by echocardiographic parameters under resting conditions. Overall, heart function did not differ between CG and TG. This is in contrast to a recent study on mice inoculated with colon-26 adenocarcinoma cells by Tian and co-workers [17] who reported a significant decrease in fractional shortening in tumor-bearing mice compared to control mice. However, their study also showed a different myocardial morphology including fibrosis and disorganization of mitochondrial cristae which was not observed in the present study. Therefore, there seem to exist general differences in the animal models between their and our study. In accordance with other studies using the Lewis lung carcinoma [18], we observed a systemic pro-inflammatory condition as characterized by elevated serum levels of Il-6 and TNF-α. In human cancer cachexia, a variety of cytokine levels is elevated with TNF-α being the key cytokine [19]. The excess cytokines may originate both from tumor cells and from immune cells being stimulated by direct or indirect signals of the tumor [19]. Pro-inflammatory cytokines, particularly TNF-α, are known to interact with the nervous system and thus may influence neuronal metabolism and survival [20]–[22], however, our data do not provide a cause-effect relationship between inflammation and the neuronal phenotype as other pathophysiological events are present in the tumor cachexia model that might affect the myocardial innervation (e.g. weight loss).

The key findings of the present study are a reduction in the myocardial innervation and a decrease in LDCV per axon profile to approximately 50% of control values. These morphological data are well supported and complemented by the qRT-PCR analysis which demonstrates a decreased expression of NPY in the neurons of the stellate ganglion while the mRNA levels of tyrosine hydroxylase are enhanced in TG. In addition, NGF mRNA expression levels were reduced in heart and stellate ganglion. The decrease in the total length of axons ramifying between cardiomyocytes is indicative of atrophic changes in the autonomic nervous system of the heart. This new finding confirms our hypothesis and strongly supports the involvement of the cardiac autonomic nervous system in cancer disease. A reduction in the total length of axons may originate i) from a loss of neurons in the ganglion, ii) from a loss of arborisation of each neuron or iii) from the loss of individual axon length. Our study shows that the reduction of the total axon length is mainly due to a decrease in nerve fiber density, not because of a reduced number of axons per nerve fiber. However, the decreased nerve fiber density was observed throughout the whole ventricle without patches of complete denervation. Thus, the result may either originate from a degenerative process that starts in the periphery of the axon branches or is based on a structural and/or metabolic affection of the nerve cell body. Studies in homozygous mice with null mutations in the NGF or the NGF receptor tyrosine kinase gene have demonstrated that the maintenance of the neurons and their arborisation in the sympathetic nervous system depends on the supply with NGF from the target tissues [23]. The reduced expression of NGF in heart and stellate ganglion makes it very likely that a lack of NGF plays a causal role in the reduction of myocardial innervation. Interestingly, there is also evidence that the metabolic functions of the neurons are disturbed as indicated by the reduction in the number of LDCV and the reduced NPY mRNA expression. In contrast to catecholamine-containing small vesicles, these LDCV contain neuropeptides which are synthesized in the soma of the neurons and are anterogradely transported to the periphery of the axons. In the heart, LDCV mainly contain NPY which has vasoconstrictory effects and serves trophic functions on cardiomyocytes [24]. It induces cardiomyocyte protein synthesis [25] and stimulates hypertrophy [26]. The reduction of NPY-containing LDCV may at least partly be responsible for the reduced content of myofibrils inside the cardiomyocytes. Interestingly, although NPY was thought to play a major role in anorexia and cachexia based on its central reduction in these conditions [16], measurements of NPY plasma levels in human control and cancer patients did not reveal differences [27]. Nevertheless, it is still debated whether influencing this system may attenuate cancer cachexia [28]. The main neurotransmitter of the sympathetic neurons (noradrenalin) is synthesized from tyrosine through various steps that are enzymatically catalyzed. The rate-limiting enzyme, tyrosine hydroxylase, was found to be upregulated in the stellate ganglion in TG which seems to be a counter-regulatory mechanism to the reduced innervation of the myocardium. The reduction of axon branches in the left ventricle may therefore be counterbalanced by enhanced catecholamine production and thus, may explain why we did not detect any functional differences under resting conditions. On the other hand, a completely denervated heart presents a very constant beat frequency but no functional impairment under rest [29] so that functional changes associated with loss of or disturbed myocardial innervation may rather become apparent under stress conditions or by analysis of specific parameters such as heart rate variability. Unfortunately, due to the complexity of the model (cardiomyocyte atrophy, skeletal muscle atrophy, anemia) it is hardly possible to perform a stress test that would clearly distinguish between changes in myocardial innervation and other extracardiac facors.

The cardiomyocyte compartment in the tumor mice was mainly characterized by a reduction of myofibrils, and an increase in sarcoplasm and lipid droplets. Investigations of the pathology of human myocardium, obtained from hearts of patients who had died from cancer cachexia, had demonstrated decreased cross-sectional area of cardiomyocytes and an increase in lipofuscin granules [2], [3]. Partly due to species differences, these results differ from those obtained in the present study. For example, in contrast to humans where age is related with an increasing volume of lipofuscin, this does not occur to a great extent in murine cardiomyocytes, probably due to the short life span of mice. The decreased cross-sectional area of cardiomyocytes as described by Burch et al. [2] is indicative of cardiac atrophy. However, in our model there was no decrease in the total volume or number of cardiomyocytes. This may be due to a more advanced disease state in the human hearts, to the time course between disease begin and analysis (21 days in mice versus months or years in humans) and of course due to differences in the complexity of the disease (no metastasis in the mouse, only mild cachexia). In rabbits subjected to cancer cachexia, there were no apoptotic cardiomyocytes in contrast to other organs where a large amount of cells underwent apoptosis [30]. At the ultrastructural level, however, a loss of myofibrils was observed which was accompanied by an increase in sarcoplasmic volume. The decrease in myofibrils is indicative of an imbalance between protein degradation and synthesis favouring protein catabolism [5]. Because of the unchanged volume of cardiomyocytes the changes in myofibrillar/sarcoplasmic volume cannot be induced by cellular edema. An interesting structural observation was the increase in lipid droplets which was confirmed by biochemical measurements of triglycerides. In tumor cachexia, fatty acids are released from the adipose tissue and accumulate in other cell types where they may cause lipotoxic effects [31], [32]. Cardiac lipotoxicity involves the generation of reactive oxygen species and the onset of apoptotic pathways [33]. In the current analysis, lipid peroxidation was assessed by measuring malondialdehyde and associated compounds, and apoptosis was evaluated by a caspase activity assay and morphological evaluation. Since none of these parameters was significantly different between TG and CG, we conclude that the observations of this study are not related to lipotoxicity. Although the exact functional significance of the observed ultrastructural alterations of the cardiomyocytes remains to be investigated, it is very likely that these changes are in direct relationship to the lack of trophic NPY stimulus via the NPY Y5 receptor [34] and may become functionally relevant if the metabolic demand of the heart increases. Due to the relatively large cardiac plasticity these changes may not necessarily lead to a significant impairment of cardiac function [35].

In summary, this study for the first time demonstrates that cancer cachexia is associated with marked structural alterations of the autonomic innervation of the heart, including both impairment of the structural integrity of the neuron (diminished total axon length) and organelle loss. Concomitantly, the amount of lipid and the number and volume of lipid droplets increased in cardiomyocytes and myofibrillar volume decreased, which is in line with the concept of a continuous trophic support of cardiomyocytes by autonomic neurons.

## Materials and Methods

### Ethics Statement

All the animal experiments were carried out in strict accordance with the recommendations in the Guide for the Care and Use of Laboratory Animals of the National Institutes of Health. The protocol (GZ 66.010/0085-II/10b/2009) was approved by the Austrian committee on the ethics of animal experiments (Bundesministerium für Wissenschaft und Forschung).

### Animals

Female C57BL/6J mice were maintained on a regular light-dark cycle (12 h light, 12 h dark) and kept on a standard laboratory chow diet (4.5% w/w fat). Mice used for injection of tumor cells as well as control animals were 8–9 weeks of age. During experiments food intake as well as body weight were monitored daily.

### Tumor implantation

Mice were randomly divided into two groups: control non-tumour-bearing mice (CG, n = 18) and mice that were injected with Lewis lung carcinoma cells (TG, n = 18). Carcinoma cells were maintained in cell culture in DMEM high glucose medium supplemented with 10% (v/v) FBS, 2 mM L-glutamine and 1% penicillin-streptomycin. Subsequently, 2×10^6^ LLC cells, obtained from an exponentially proliferating cell-culture, were subcutaneously injected below the animal's neck. Sibling mice were injected with PBS at the same place to serve as sham controls. Both control and tumor bearing mice were sacrificed after 21 days. In separate sets of experiments, the mice were used (i) for echocardiography (n = 4 in tumor group, n = 5 in control group) and stereology (n = 5 in tumor group, n = 6 in control group), (ii) for measuring cardiac lipid content, caspase activity, lipid peroxidation and serum cytokine levels (n = 6 in each group), and (iii) for measuring body, left ventricular and tumor weight and for polymerase chain reaction of left ventricle and stellate ganglion (n = 6 in each group). Differences in the number of animals used for echocardiography and stereology are caused by exclusion of animals that stopped breathing spontaneously during anesthesia (echocardiography) or that were not properly fixed by vascular perfusion (stereology).

### Echocardiography

2D guided M-mode echoes (30 MHz) were obtained from short- and long-axis views at the level of the largest LV-diameter using a VS-VEVO 770 High Resolution Imaging System (Visualsonics, Toronto, Canada) equipped with a 30 MHz RMV (Real time microvisualization) scan head. Mice were lightly anesthetized with 2% isoflurane and were allowed to breathe spontaneously. The chest was shaved, acoustic coupling gel was applied, and a warming pad was used to maintain normothermia. Mice were imaged in a shallow left lateral decubitus position. Left ventricular end-diastolic and end-systolic dimensions were measured from original tracings by using the leading edge conventions of the American Society of Echocardiography. Left ventricular percent fractional shortening was calculated as previously described [36].

### TNF-α and Il-6 ELISA

Blood samples were taken from anesthetized control and tumor-bearing mice by retro-orbital puncture. The serum was frozen at −20°C. Serum levels of TNF-α and Il-6 were measured using TNF-α ELISA ReadySETGo (88-7324, eBiosciences, San Diego, USA) and Mouse Il-6 ELISA ReadySETGo (88-7064), eBiosciences, San Diego, USA). The plate was finally read at 450 nm.

### Triglyceride measurement

Triglycerides were extracted from cardiac tissues of control and tumor-bearing mice using hexane∶isopropanol and saponified using 1% triton-x dissolved in chloroform. The organic solvents were evaporated using N_2_ gas and the triglyceride content was measured using Triglyceride FS kit (Diasys, Germany) according to the supplier's manual. The triglyceride levels were normalized to the tissue weight.

### Measurement of lipid peroxidation and caspase activity

To evaluate the presence of lipotoxic effects on cardiomyocytes, lipid peroxidation was determined by measuring malondialdehyde and apoptosis was determined by measuring caspase activity.

Cardiac tissue was washed thoroughly with heparin-PBS solution containing 1 mM EDTA and homogenized in 500 µl of homogenization buffer (0.25 M sucrose, 1 mM EDTA, 1 mM DTT, 1 mM deferoxamine mesylate, 1 mM PMSF) at 4°C. Protein concentration was determined using RC-DC protein assay (Bio-Rad, Hercules, CA, USA) from the supernatant. Lipid peroxidation was measured in the supernatant using the Oxiselect MDA adduct ELISA kit (STA-332, Cell Biolabs, San Diego, CA, USA) and TBARS assay (STA-330, Cell Biolabs) according to the manufacturer's protocol.

For measurement of caspase 3/7 activity, 15 to 25 mg cardiac tissue were homogenized in 300 µl of homogenization buffer (25 mM Hepes pH 7.5, 5 mM EGTA, 1 mM Pefabloc SC, 1% protease inhibitor cocktail [v/v]) (P8340, Sigma Aldrich, St. Louis, MO, USA) at 4°C. The lysate was centrifuged at 13,000 g for 20 min at 4°C. The supernatant was collected and the protein content was measured using the DC Protein Assay (Bio-Rad, Hercules, CA, USA). Caspase activity was determined by incubating 50 µg protein in 100 µl of Caspase-Glo®3/7 reagent (Promega, Madison, WI, USA) for 1.5 hours at 20°C and luminescence was determined using a luminometer (LUMIstar Optima BMG Labtec, Offenburg, Germany).

### Quantitative RT-PCR

Total RNA was extracted from the hearts and stellate ganglia of control and tumor-bearing mice (n = 6 each) by the Trizol reagent (Invitrogen, Carlsbad, California, USA) according to the manufacturer's protocol. One microgram of total RNA was transcribed reversely into cDNA using the high capacity cDNA reverse transcription kit (Applied Biosystems, Carlsbad, CA, USA). Quantitative real-time polymerase chain reaction (qRT-PCR) was performed using gene specific primers purchased from MWG-Biotech (Ebersberg, Germany) (**Table 6**). 18 s RNA was used as internal control. All reactions were carried out in triplicates. Quantitative RT-PCR was performed on a 7900T standard rea-ltime PCR system (Applied Biosystems) using SYBR green as the detection flurophore. The results are presented as relative expression levels after calculation based on the delta delta C_T_ method [37].

**Table 6:** Primers used for quantitative RT-PCR.

Target	Forward primer	Reverse primer
TNF-R1	CGGAAAGAAATGTCCCAGGT	GCGTTGGAACTGGTTCTCCT
TNF-R2	CCTCCTGGCCAATATGTGAA	ACATGCTTGCCTCACAGTCC
β1-R	CTGCTACAACGACCCCAAGT	GGCACGTAGAAGGAGACGAC
NGF	CATGGGGGAGTTCTCAGTGT	TTGATGTCTGTGGCTGTGGT
NPY	CATGTGGTGATGGGAAATGA	GGTTTCAGGGGATGAGATGA
TH	TCAGAGCAGGATACCAAGCA	GGGCATCCTCGATGAGACT

Legend. TNF-R1 = tumor necrosis factor receptor 1, TNF-R2 = tumor necrosis factor receptor 2, β1-R = β1 adrenergic receptor, NGF = nerve growth factor, NPY = neuropeptide Y, TH = tyrosine hydroxylase.

### Tissue fixation, sampling and processing

After echocardiography, mice were anticoagulated with 500 IE heparin i.p., anesthetized with isoflurane and killed by cervical dislocation 3 min later. The heart was then excised, mounted in a Langendorff apparatus, perfused with 4% paraformaldehyde in phosphate buffer retrogradely via the aorta for 10 min and kept in paraformaldehyde at 4°C until further processing. The duration between fixation and processing was equal for all animals investigated, thus the potential effect of paraformaldehyde fixation on immunoreactivity was the same in the control and the experimental group. The left ventricle including the interventricular septum was separated from the rest of the heart and weighed. The volume of the left ventricle was calculated by dividing the mass by the density of muscle tissue, 1.06 g/cm^3^ [38]. The isolated left ventricle was sampled for microscopy as described previously [39]. In short, eight tissue samples were generated and allocated for estimation of tissue volume shrinkage, light microscopic or electron microscopic examination by systematic uniform random sampling. As several of the stereological analyses performed in this study require the use of isotropic uniform random samples, the orientator [40] and the isector method [41] were used to disorientate the samples for electron and light microscopy, respectively, and give every orientation of the heart an equal chance of being selected for the analysis.

The tissue samples for light microscopy were embedded in paraffin according to standard protocols. The tissue samples for transmission electron microscopy were osmicated, stained en bloc in half-saturated aqueous uranyl acetate, dehydrated in an ascending ethanol series and finally embedded in epoxy resin.

### Stereological estimations

**Figure 4:**
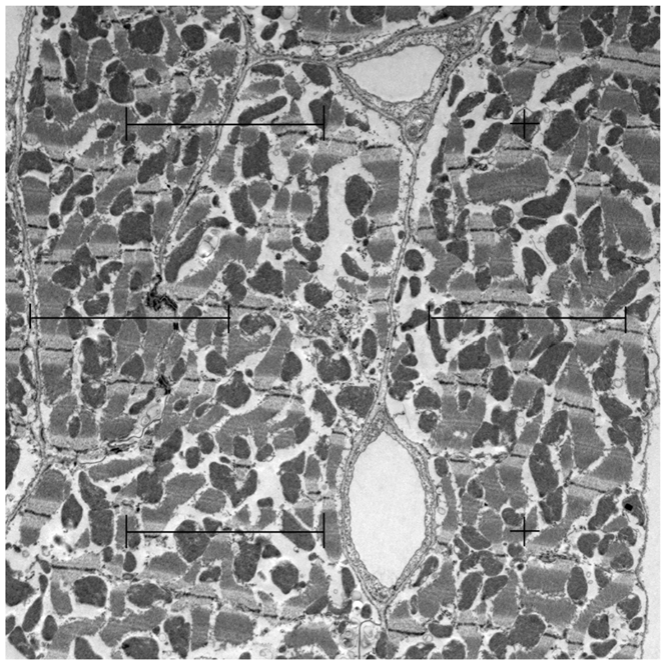
Demonstration of the point counting method. A multi-purpose grid with 10 points (crosses or endpoints of test lines) is projected on an electron micrograph. The number of points hitting a structure of interest, e.g. mitochondria (here, 2), is divided by the total number of points hitting cardiomyocytes (here, 9) to provide the volume fraction of mitochondria within cardiomyocytes (here, 2/9 = 22.22%). The volume fraction is further multiplied by the reference volume, viz. the total volume of cardiomyocytes, to provide the total volume of mitochondria in the left ventricle.

The stereological methods used in this study are well-established and have been the subject of recent reviews [42], [43]. Details on section thickness, staining and magnification are summarized in **Table 7**. For light microscopic stereology, an Olympus BX51 microscope (Olympus, Hamburg, Germany), equipped with a digital camera and the newCAST stereology software (Visiopharm, Denmark) was used. Transmission electron microscopy was performed using a LEO 902 electron microscope (Zeiss, Oberkochen, Germany). All fields of view for stereological analysis were obtained by systematic uniform random sampling [44]. The volume densities of cardiomyocytes and interstitium were estimated using light microscopy, the volume density of subcellular cardiomyocyte compartments were estimated by eletron microscopy. All volume densities were estimated by the point counting method (Figure 4) [45]. Briefly, the number of points hitting a structure of interest (P(str)) was divided by the number of points hitting the reference volume which provides the volume density (or fraction) of the structure of interest within the given reference volume. The total volume of each compartment was calculated by multiplication of the density by the respective reference volume. For example, the total volume of mitochondria within cardiomyocytes is calculated by multiplying the volume fraction of mitochondria with the volume fraction of cardiomyocytes and the volume of the left ventricle.

**Table 7:** Methodological details of stereological parameters.

Parameter	Probe	Section	Staining	Magnification
V_V_(myo/lv), V_V_(int/lv)	Test points (16 points per FOV)	Semithin section	Richardson's stain	×40
V_V_(mit/myo), V_V_(mf/myo), V_V_(sp/myo), V_V_(nuc/myo)	Test points (12 points per FOV)	Ultrathin section	Uranyl acetate & Lead citrate	×4,400
V_V_(ld/sp)	Test points (256 points per FOV)	Ultrathin section	Uranyl acetate & Lead citrate	×20,000
N_V_(nuc/lv)	Optical disector (h = 3 µm)	40 µm thick paraffin section	Haematoxylin & Eosin	×100
N_N_(nuc/myo)	Optical disector (h = 3 µm)	40 µm thick paraffin section	Wheat germ agglutinin, Immunostaining for N-Cadherin	×40
L_V_(nf/lv)	Counting frame (Area: 3,000 µm^2^)	7 µm thick paraffin section	Immunostaining for PGP 9.5	×40
Q_Q_(ax/nf)	Counting frame (Area: 20 µm^2^)	Ultrathin section	Uranyl acetate & Lead citrate	×20,000
Tissue volume shrinkage	Cavalieri method (a(p) = 1.004 mm^2^, 100 points)	Serial 7 µm thick paraffin sections	Methylene blue	×1.25

Legend. V_V_ = volume density, N_V_ = numerical density, L_V_ = length density, myo = cardiomyocytes, int = interstitium, lv = left ventricle, mit = mitochondria, mf = myofibrils, sp = residual sarcoplasm, nuc = nucleus, ld = lipid droplets, N_N_(nuc/myo) = mean number of nuclei per myocyte, nf = nerve fiber, Q_Q_(ax/nf) = mean number of axon profiles per nerve fibre profile, FOV = fields of view, h = height, a(p) = area per point.

The estimation of the total number of cardiomyocytes was based, in principle, on the method described by Brüel and Nyengaard [46]. In short, the numerical density of cardiomyocyte nuclei was estimated using the optical disector method [47] and the mean number of nuclei per myocyte was estimated from 40 µm thick paraffin sections immunostained for N-Cadherin (primary antibody: mouse-anti-pan-cadherin, 1∶4000, Sigma; secondary antibody: peroxidase-labeled goat-anti-mouse IgG) and peroxidase-labeled wheat germ agglutinin (WGA-Peroxidase, 1∶6400, Vector Laboratories, Burlingham, Canada) and subsequent 3,3-diaminobenzidine reaction to visualize the borders of cardiomyocytes [48]. Cardiomyocytes were sampled for this analysis based on the number of their nuclei using the optical disector in the middle of the section. If a cardiomyocyte was sampled, the microscope stage was moved through the whole cell in the z-direction and the number of nuclei was noted. From the total number of nuclei and the mean number of nuclei per cell, the total number of cardiomyocytes was calculated according to ref. [46].

**Figure 5:**
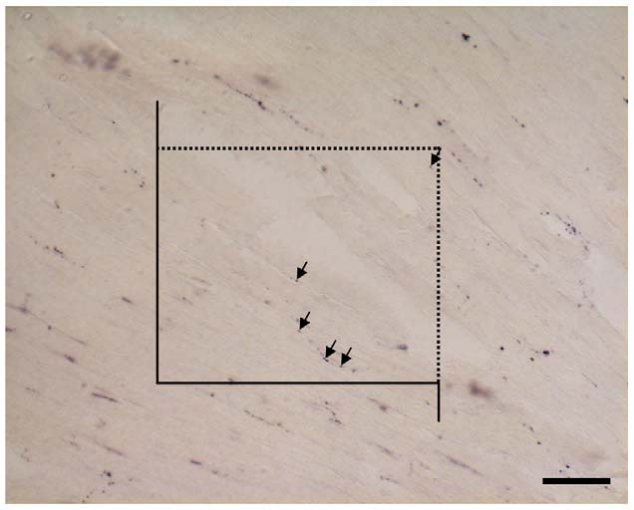
Immunohistochemistry of nerve fibers. Nerve fibers were detected by immunostaining for protein gene product 9.5 (PGP9.5). Scale bar = 50 µm. Nerve fibers (arrows) were counted in an unbiased counting frame. The dashed line is the inclusion line, the closed line and its extensions represents the exclusion line (see Methods section).

The total length of axons ramifying between cardiomyocytes was estimated by a recently established method [49]. Nerve fiber profiles were visualized by immunohistochemistry for protein gene product 9.5 (PGP9.5) [50] and counted if they were lying inside the area of an unbiased counting frame (**Figure 5**) [51] to obtain the length density of nerve fibers. According to the stereological equation L_V_ = 2*Q/A, the length density can be calculated from the number of nerve fibre profiles (Q) and the reference area used for counting (A). The latter is calculated by multiplying the area of the counting frame by the number of counting frames. At the electron microscopic level, systematic uniform randomly sampling was performed to gather images of nerve fibres (e.g. Figure 3 E and F). In these images, the number of axon profiles within one nerve fibre profile was counted and the arithmetic mean number of axon profiles per nerve fiber profile was calculated. This parameter was multiplied with the total length of nerve fibers to obtain the total length of axons in the ventricle. Two additional morphometric parameters for characterization of the myocardial innervation were determined: The mean diameter of axons was measured as the largest diameter orthogonal to the longest axon profile diameter, and the number of LDCV per axon profile was counted.

Some of the stereological parameters were obtained from paraffin embedded sections. During paraffin embedding a high and unpredictable degree of tissue shrinkage occurs and leads to false estimations if the densities obtained from the sections are simply multiplied by the reference volume before embedding [52]. Therefore, from each animal, one tissue block of left ventricular myocardium was carefully weighed before embedding and its volume was calculated. Afterwards these tissue blocks were separately embedded in paraffin and entirely cut into 7 µm thick sections, and each 10th section was mounted on a glass slide. Using the Cavalieri principle [44], the area of these sections was estimated, multiplied by the section thickness and the total number of sections. This microscopically determined volume after embedding and the volume measured before embedding were used to calculate the tissue shrinkage due to embedding. A factor correcting for tissue shrinkage was therefore included where necessary. No such correction was done for samples embedded in Epon because previous studies had shown that tissue shrinkage in epoxy resin is less than 5% [39], [52].

### Statistics

All data are given as mean ± standard deviation in the tables or as individual values in the figures. Control and tumor bearing mice were compared using the nonparametric two-sided Mann-Whitney U-test for independent samples. Differences were considered statistically significant if p<0.05.
